# Rutin of *Moringa oleifera* as a potential inhibitor to *Agaricus bisporus* tyrosinase as revealed from the molecular dynamics of inhibition

**DOI:** 10.1038/s41598-024-69451-y

**Published:** 2024-08-29

**Authors:** Reyad M. El-Sharkawy, Abdalla E. El-Hadary, Heba S. Essawy, Ashraf S. A. El-Sayed

**Affiliations:** 1 Botany and Microbiology Department, Faculty of Science, Benha University, Benha, 13511 Egypt; 2 Biochemistry Department, Faculty of Agriculture, Benha University, Benha, Egypt; 3 Enzymology and Fungal Biotechnology Lab (EFBL), Botany and Microbiology Department, Faculty of Science, Zagazig University, Zagazig, 44519 Egypt

**Keywords:** *Agaricus bisporus*, Tyrosinase, Inhibition, Kinetics, Flavonoid, Rutin, Kojic acid, Anti-pigment, Cosmetics, Biocatalysis, Enzyme mechanisms, Enzymes, Structural biology, Biochemistry

## Abstract

Tyrosinase is a binuclear copper-containing enzyme that catalyzes the conversation of monophenols to diphenols via *o*-hydroxylation and then the oxidation of *o*-diphenols to *o*-quinones which is profoundly linked to eukaryotic melanin synthesis and fruits browning. The hyperpigmentation due to unusual tyrosinase activity has gained growing health concern. Plants and their metabolites are considered promising and effective sources for potent antityrosinase enzymes. Hence, searching for potent, specific tyrosinase inhibitor from different plant extracts is an alternative approach in regulating overproduction of tyrosinase. Among the tested extracts, the hydro-alcoholic extract of *Moringa oleifera* L. leaves displayed the potent anti-tyrosinase activity (IC_50_ = 98.93 µg/ml) in a dose-dependent manner using _L_-DOPA as substrate; however, the kojic acid showed IC_50_ of 88.92 µg/ml. The tyrosinase-diphenolase (TYR-Di) kinetic analysis revealed mixed inhibition type for the *Ocimum basilicum* L. and *Artemisia annua* L. extracts, while the *Coriandrum sativum* L. extract displayed a non-competitive type of inhibition. Interestingly, the extract of *Moringa oleifera* L. leaves exhibited a competitive inhibition, low inhibition constant of free enzyme (${\text{K}}_{\text{ii}}^{\text{app}}$) value and no Pan-Assay Interfering Substances, hinting the presence of strong potent inhibitors. The major putative antityrosinase compound in the extract was resolved, and chemically identified as rutin based on various spectroscopic analyses using UV–Vis, FTIR, mass spectrometry, and ^1^H NMR. The in silico computational molecular docking has been performed using rutin and *A. bisporus* tyrosinase (PDB code: 2Y9X). The binding energy of the predicted interaction between tropolone native ligand, kojic acid, and rutin against 2Y9X was respectively − 5.28, − 4.69, and − 7.75 kcal/mol. The docking simulation results revealed the reliable binding of rutin to the amino acid residues (ASN^260^, HIS^259^, SER^282^) in the tyrosinase catalytic site. Based on the developed results, rutin extracted from *M. oleifera* L. leaves has the capability to be powerful anti-pigment agent with a potential application in cosmeceutical area. In vivo studies are required to unravel the safety and efficiency of rutin as antityrosinase compound.

## Introduction

Tyrosinase (EC 1.14.18.1) is a critical key enzyme in different physiological processes, particularly correlated to rate-limiting step in the pigment synthesis^1–3^. Tyrosinase catalyzes vegetable browning^4^, pigment synthesis in mammals^5^, insect cuticle production^6^, and healing of wounds^7^. This enzyme belongs to a metalloprotein-polyphenol oxidase with the H_2_L_2_ tetramer structure in which 392 amino acid residues and 2 Cu ions are in the 2H subunits and 150 amino acid residues in 2 L subunits^8,9^. It can perform bifunctional activities including monophenolase and diphenolase activity. The monophenolase (cresolase) activity catalyzes the conversation of monophenols to diphenols via *o*-hydroxylation. While the diphenolase (catecholase) activity catalyzes the passage of *o*-diphenols to *o*-quinones via oxidation^10,11^.

Melanin is a ubiquitous natural pigment which is related to the color of skin, hair, and eyes of humans. It is responsible for the skin fortification against harmful effects of ultraviolet irradiation^5,12^. The synthesis of melanin pigment (melanogenesis) is initiated by a regulating step in which tyrosinase can convert _L_-tyrosine to l-DOPA (l-3,4-dihydroxyphenylalanine) which is then oxidized to dopaquinone and finally produce pheomelanine and eumelanine^8,11,13,14^. Pigmentary disorders due to unusual tyrosinase activity causes hyperpigmentation and in turn dermatological disorders including melasma, melanoderma, freckles, and ephelides^15,16^.

The skin lightening products have been reported by current meta-analysis to be widely employed amongst African people^17^. High prevalence of such materials creates a motivation of science toward cosmetics platform associated with health concern^5,14^. Interestingly, tyrosinase inhibitors are considered as an efficient approach for controlling the abnormal melanin accumulation and browning in fruits^11,18,19^. Skin lighteners have a great impact in the cosmetic industry due to their antityrosinase activity. They can act using various action mechanisms including inhibition of enzyme transcription, transfer of melanosome, bind to tyrosinase active site, and enhance turnover of epidermal cells^20,21^.

Different natural and synthetic materials with potent antityrosinase inhibitory activity have been explored; however, clinical trials should be carried out to investigate their effectiveness as antityrosinase agents^11,12,14,22^. Among them, hydroxyphenolic compounds, hydroquinone, tropolone, steroids, kojic acid, and their derivatives are commonly used as anti-browning and antityrosinase agents^5,9,11^. The main drawbacks of these compounds are related to their low efficiency, low activity, less safety, mutagenesis, and carcinogenesis; hence, it is obligatory to explore a powerful specific antityrosinase ingredient with less undesirable effect.

Antityrosinase ingredients of natural and plant resources are much safer, and less toxicity, comparing with synthetic ones^16^. Plants are considered as one of the most vital sources for various natural bioactive phytochemical molecules^5^. In traditional medicines, different plant extracts are employed for skincare and control of hyperpigmentation disorders due to their constituents of oils, antioxidants, vitamins, phenolics, flavonoids and other constituents of biological importance^5,15^. Of which, *Moringa oleifera* L. belongs to the family Moringaceae, is commonly found in Africa and India^23^. Flavonoid constituents are the most abundant polyphenols in different plant parts including flowers, leaves, bark, and seeds^21,24^. These constituents including flavonols, anthocyanidins, flavones, isoflavones, and flavanones can perform several biological activities such as anticancer, antioxidant, anti-inflammatory, and anti-genotoxic that enhance its promotional properties as previously reported^5,24–27^. The incorporation of various bioactive constituents within plant extracts in inhibitory process of tyrosinase causes an imprecision in the inhibitory mechanism and hence reduces its practical application^27,28^. Therefore, continuous searching for more selective inhibitors is of desirable need with emphasis on their inhibition mechanism and kinetics. A variety of tyrosinase inhibitors namely uncompetitive, competitive, noncompetitive, and mixed inhibitors, have been used to study the kinetics of inhibitors^3,4,15,24,28^. Compared to control (without inhibitor), competitive inhibitors are able to bind only to the free enzyme and not enzyme–substrate complex, assuming a reduction in the kinetic constants (K_m_ and V_max_) which is reversible by addition of more substrate^28^. The kinetic study is frequently preferred to be performed on the diphenolase activity of tyrosinase rather than monophenolase activity. This is not only due to the necessary to eradicate the delay period (lag period) to achieve steady state in monophenolase reaction, but also the diphenolase activity is normally behaves as Michaelian type and hence enables the characterization of inhibitory type and the inhibitor strength^2,24,29,30^.

Rutin is the highest prominent and explored flavonoids in *Moringa oleifera* L.^31^. It’s composed of quercetin (flavonol) 3-*O*-bind to rutinose (disaccharide). Interestingly, the natural quercetin-glycosides (rutin) displayed a potential antityrosinase, anticancer, anti-inflammatory activities and can improve in vivo wound healing^5,12,23,32^. Based on these findings, rutin is reported as an interesting flavonoid compound for clinical applications; however, its low bioavailability, poor absorption and water solubility is the main limitations for its broad applications^33,34^. To reduce cost and time, virtual docking simulation has been performed in the primary steps by researchers to discover the interaction between proteins and various ligands^5,32^. The crystal structure of tyrosinase has been well known recently and hence enables us to evaluate a characteristic insight the interaction mechanism between tyrosinase catalytic site and different ligand compounds^8,16^. The 3D-structure of tyrosinase revealed the protein pocket has amino acid residue coordinated with a binuclear Cu site. The first Cu ion coordinates with histidine residues (HIS^85^, HIS^61^, and HIS^94^) and the second Cu ions displayed a coordination with HIS^263^, HIS^259^, and HIS^296^^5^.

The novelty of the current study was to obtain antityrosinase compound from the plant sources with a lower binding energy, higher stability, and higher bond strength against the target protein, compared to the authenticate inhibitor. The objective of this work was to evaluate the tyrosinase inhibitory kinetics by various selected plants extracts with a correlation to their flavonoid contents. The unveiling of bioactive constituents in a selected indigenous African plant with high flavonoid constituents and a promising antityrosinase activity have been carried out by HPLC profile analysis. The putative predominant flavonoid compound (rutin) was isolated, and purified from *Moringa oleifera* L. leaves, and then subjected to spectroscopic analyses including UV spectrophotometer, FTIR, mass spectrum, and ^1^H NMR to confirm the chemical identity of rutin. To predict the binding mechanism of rutin against *Agaricus bisporus* tyrosinase (PDB code of 2Y9X), in silico computational molecular docking method was performed using Molecular Operating Environment (MOE, 2016)^35^ program version 10.

## Materials and methods

Tyrosinase from *A. bisporus* (2Y9X, EC 1.14.18.1, T3824), kojic acid, _L_-dihydroxyphenylalanine (_L_-DOPA), 2,2-diphenyl-1-picrylhydrazyl (DPPH), were of analytical grade and obtained from Sigma-Aldrich Chemical Co (USA). Dimethyl sulfoxide (DMSO, 5%) was employed for rutin dissolving into solution. Methanol, *n*-hexane, and ethanol were of analytical grade. Silica gel (100–200 mesh) was employed for analytical column chromatography and was purchased from Merck. Other chemicals and reagents were grade quality and purchased from Sigma-Aldrich Chemical Co (USA). In the docking modelling analysis, the tyrosinase protein from *Agaricus bisporus* (PDB code of 2Y9X) was retrieved from the Protein Data Bank (http://www.rcsb.org). The 3D-structures of the rutin, tropolone, and kojic acid have been created by using the structure regained from the database of PubChem (http://pubchem.ncbi.nlm.nih.gov). The docking procedure was performed using Molecular Operating Environment (MOE, 2016) program version 10^35^.

### Plant materials

The leaves of *Moringa oleifera* were obtained from the Horticulture Department, Faculty of Agriculture, Benha University, Egypt, and all methods were performed according to the national guidelines, and the samples were identified^36^. Specimens of the plant were deposited in the Botany Department Herbarium, Faculty of Science, Benha University, Egypt, under deposition # FS-BU-1-2023.

### Preparation of plant extract

The plant extracts were prepared by carefully washing of the healthy fresh leaves, air drying at the ambient temperature, homogeneously pulverizing to a fine powder by a blender^27^. The powders (1:4, w/v) were separately extracted by using absolute methanol and stirred at room temperature for 6 h. The resulting extracts were separately filtered by Whatman® no. 1 filter paper, and the supernatant was gathered after centrifugation at 10,000×*g* for 15 min. The previous procedure was repeated three times using fresh methanol solvent. The extracts were then independently concentrated under vacuum using rotary evaporator at 50 °C, freeze-dried, and stored in the dark at 2 °C for further use^25^.

### Determination of total flavonoid content

The total flavonoid contents (TFC) in the investigated plant extracts were determined by using the aluminum chloride^37^. In brief, the extract of each sample (0.5 ml) was separately mixed with deionized water (1.5 ml), NaNO_2_ (0.1 ml, 5% solution) and AlCl_3_ (0.1 ml, 10% w/v). The preparations were incubated at 25 °C for 5 min, followed by addition of NaOH (2 ml, 4% solution) and distilled water to be 5 ml in total volume. After 15 min in dark, the absorbance was monitored at 510 nm. The concentration of TFC was represented as mg quercetin equivalent (QE)/g dry weight^38^.

### Tyrosinase inhibition assay

Tyrosinase activity was spectrophotometric assayed as illustrated previously^3,4,9,32^ with slight modification. Enzyme activity was investigated via the formation of colored dopachrome. In this method, the examined samples (plants extract) were firstly prepared in DMSO and subsequently diluted to the required concentration using phosphate buffer (pH 6.8). In 96-well microtiter plates, the preparations were contained 50 µl *A. bisporus* tyrosinase in phosphate buffer (100 mM, pH 6.8) and 50 µl of various concentrations of the tested samples. After pre-incubation at 25 °C for 15 min, 50 µl of l-DOPA (15 mM) in phosphate buffer (100 mM, pH 6.8) solution was added to the mixture. After incubation in dark place for 30 min, the absorbance was examined at 492 nm by investigating the amount of dopaquinone formed. Kojic acid and DMSO without examined material were prepared as mentioned before and then assessed under similar conditions and used as positive and negative controls, respectively.

The enzymatic activity in absence of inhibitor was defined as 100%. The inhibition activity of the investigated compound toward tyrosinase was assessed using the following equation:

1 $$\text{Tyrosinase inhibition }\left(\text{\%}\right)=\frac{{A}_{C}- {A}_{T}}{{A}_{C}}\times 100$$

where ${A}_{C}$ and ${A}_{T}$ are the absorbance value without inhibitor (control) and with the tested sample (inhibitor).

The tyrosinase inhibition percentages were plotted versus the concentration of inhibitor. A linear fitting equation have been obtained and then the tyrosinase inhibition by using the tested plants was expressed as IC_50_, representing the concentration of the tested extract that inhibit 50% of the initial enzymatic activity.

### Kinetic assessment of tyrosinase inhibition

#### Determination of the inhibition type

To determine the inhibition kinetics of *A. bisporus* tyrosinase by the plant extracts, a sequence of experiments was accomplished using l-DOPA as substrate in the absence or presence of inhibitor. Briefly, the reaction mixture contained various concentrations of separately plant extracts (50–150 µg/ml), 100 µl phosphate buffer (100 mM, pH 6.8), and 50 µl l-DOPA. *A. bisporus* tyrosinase (50 µl) was added to initiate the reaction, followed by incubation for 15 min at 25 °C under aerobic conditions, and the absorbance determination at 492 nm.

The kinetic constants namely, maximal velocity (V_max_) and Michaelis constant (K_m_), of tyrosinase-diphenolase were determined from primary simulation dataset of various concentrations of _L_-DOPA (0.5–6 mM) using the Lineweaver–Burk plot by Eq. (2).

2 $$\frac{1}{V}=\frac{{K}_{m}}{{V}_{max}} \frac{1}{[S]} + \frac{1}{{V}_{max}}$$

The apparent inhibition constant of free enzyme (${\text{K}}_{\text{i}}^{\text{app}}$) was assayed from the plot of the Lineweaver–Burk slopes and the extract concentrations using the Eq. (3).

3 $$\text{Slope}=\frac{{K}_{m}}{{V}_{max}} \left(1+ \frac{\left[\text{I}\right]}{{\text{K}}_{\text{i}}^{\text{app}}}\right)$$

The apparent inhibition constant of enzyme–substrate complex (${\text{K}}_{\text{ii}}^{\text{app}}$) was determined by plotting the extract concentrations versus the y-intercepts of Lineweaver–Burk plot using the Eq. (4).

4 $$\text{Intercept}= \frac{1}{{V}_{max}} (1+ \frac{[\text{I}]}{{\text{K}}_{\text{ii}}^{\text{app}}})$$

### Evaluation of antioxidant activity

The in vitro antioxidant capacity of three different extracts from *Moringa oleifera* L. leaf powder was evaluated by the DPPH and free radicals scavenging assay^39,40^. In brief, the methanolic DPPH solution was mixed at room temperature with various concentrations of the examined extract for 15 min. Control preparations containing methanolic DPPH without the tested leaves extract were performed using the previous conditions. The absorbance was monitored by spectrophotometer at 517 nm and the % DPPH scavenging inhibition was calculated based on the subsequent equation:

5 $$\text{Inhibition }\left(\text{\%}\right)= \frac{{D}_{C}- {D}_{S}}{ {D}_{C}} \times 100$$

where, ${D}_{C}$ describe the control absorbance (without extract) and ${D}_{S}$ describe the absorbance in the presence of plant extract. The values of IC_50_ which are the quantity of the examined sample require to reduce the DPPH (initial concentration) by 50%. The values of IC_50_ were calculated via a linear regression of the developed data and represented in (µg/ml).

The free radical scavenging activity of the plant extract was performed by incubating various concentrations of the extract with H_2_O_2_ solution (2 mM, 0.6 ml) and phosphate buffer (0.3 ml, 50 mM, pH 7.0) for 20 min. The reduction in absorbance of the preparation containing the plant extract was recorded at 320 nm with respect to the control (without inhibitor). The values of IC_50_ were detectable via a linear regression of the obtained data.

### Flavonoid isolation from *Moringa* oleifera leaves

The flavonoid compounds of the potent inhibitor for the tyrosinase-diphenolase activity were isolated from leaf powder of *Moringa oleifera* L., according to the protocol described by Hamed et al.^24^ and Arafa et al.^41^. The crude extract of *M. oleifera* L. leaf powder, obtained by the ultrasonic hydro-alcoholic (1:1 v/v, water : methanol) extraction, was filtered, centrifuged, and concentrated using vacuum filtration. In a separating funnel, the crude extract was further combined with n-hexane and then gathered at the upper phase. While the water phase (lower layer) was concentrated and partitioned by using ethyl acetate and n-butanol. The previous step was repeated twice, and the obtained 3 fractions were concentrated under vacuum at 50 °C using rotary evaporator and subsequently lyophilized (24 h, − 80 °C). All fractions were kept in dark and dry place at 2 °C for further use. The fractions developed from the extract of *M. oleifera* L. leaves (n-hexane, ethyl acetate, n-butanol) were then examined for their inhibitory activity against tyrosinase. The fraction which displayed the lowest value of IC_50_ against tyrosinase as illustrated by the standard assay, was selected for the putative flavonoid’s purification. The fraction of ethyl acetate from *Moringa oleifera* L. leaf powder was subjected to further fractionation for the isolation of different flavonoids using the elution solvent of hydro-alcoholic solution with 1.5 ml/min flow rate.

### HPLC analysis of the *Moringa* oleifera leaves extracts

The phytochemical composition of the hydro-alcoholic (1:1 v/v, water:methanol) extraction of *Moringa oleifera* L. leaf powder was carried out by High Performance Liquid Chromatography (HPLC) system^41^. The analysis by HPLC was performed using Agilent 1200 HPLC system (Agilent technologies, USA), operated with a quaternary pump (L2130), auto-sampler and a UV detector (L2400) at wavelengths of 254 nm, 280 nm and 320 nm. The acquisition and processing of data was performed by chemstation HPLC software. Additionally, Phenomenex BDS RP-18 column maintained at 25 °C was used for the qualitative and quantitative analyses. A 20 µl injection of methanolic extract of *M oleifera* L. leaves (10 mg/ml) was carried out to achieve the chromatogram of HPLC. A gradient mobile phase system consisted of water (acidulated with 1.4% *O*-phosphoric acid)/acetonitrile (85:13.8 v/v) with a flow rate of 1 ml/min for 20 min. The extract constituents were identified based on the peak retention time when compared with those of the available authentic standard samples.

### Rutin isolation and purification from *Moringa* oleifera leaves

The rutin was isolated by ethylacetate extraction^15,41^, the extract was fractionated with column chromatography using a Sephadex G-100 as a stationary phase and methanol as the eluting phase. The selected fraction (no. 10)-based on its high antityrosinase activity, was subjected to further partition using column chromatography packed with Sephadex G-100 with the same elution solvent. The developed sub-fractions were examined according to their antityrosinase activity. The putative rutin sample (sub-fraction no. 10-B) was loaded onto the column and eluting by the same elution solvent with a flow rate retained at 1.5 ml/min^31^. The putative rutin sample was subjected to crystallization by removing solvent via rotary evaporator. The dried preparation was mixed under heat with a little volume of water, followed by filtration and preserve at 4 °C for the development of crystals^31^.

### Chemical analysis of the extracted rutin from *Moringa* oleifera leaves

The UV spectra of the isolated compound (putative rutin) were monitored using spectrophotometer in the range of 150–700 nm. The Fourier Transform Infrared (FTIR) spectroscopy of the purified rutin sample was assessed using a Bruker spectrometer within a range of 500–4000 cm^−1^. The putative rutin was identified by recording the fragmentation spectrum in the spectral range of *m*/*z* 50–540 using mass spectroscopy with electron ionization at 70 eV and comparing the sample fragmentation results with those in NIST and WILEY mass spectral library. The chemical identity of the extracted compound was further affirmed from the ^1^H NMR spectrum using a Bruker 400 M spectrometer (Germany). The sample was primary dispersed in DMSO and then analyzed at 400 MHz. All chemical shifts were expressed in δ-part per million scale.

### In silico molecular docking of tyrosinase and rutin

**Figure 1:**
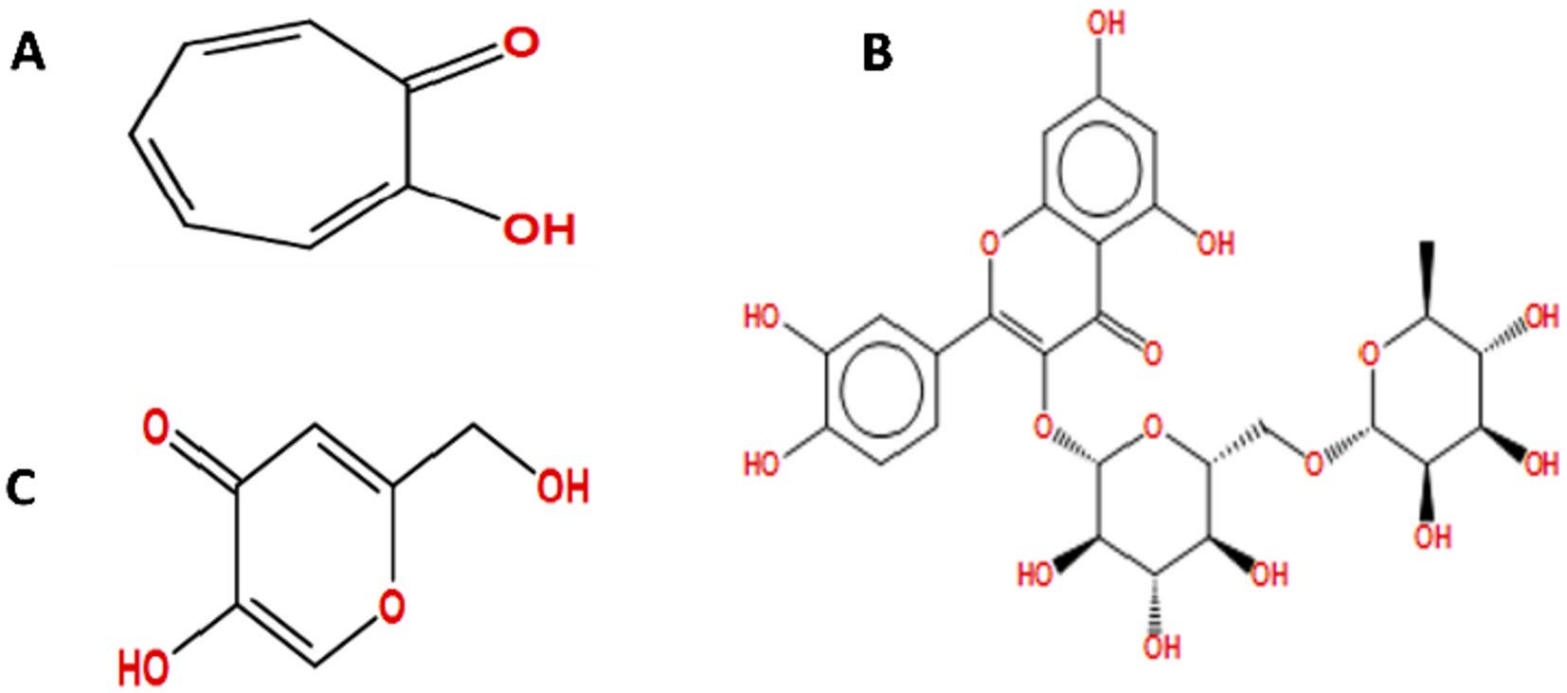
The structures of the examined tyrosinase inhibitory ligands: (**A**) tropolone (inherent ligand of target protein tyrosinase (PDB, 2Y9X), (**B**) rutin, and (**C**) kojic acid.

The crystal structure of the target protein for *Agaricus bisporus* with small inherent inhibitor (tropolone) was retrieved from the Protein Data Bank (http://www.rcsb.org) under the PDB code of 2Y9X^1,8,42^. The 3-dimentional protein structure was prepared by performing several steps: elimination of the water molecules and the native ligand, attachment of hydrogen atoms, merging non-polar hydrogens, calculation of Gasteiger charges for 2Y9X, and localization of pockets. The 3D-structure of the examined inhibitor (rutin) has been created by using the structure regained from the database of PubChem (http://pubchem.ncbi.nlm.nih.gov), and then optimized before the performance of molecular modelling. The validity of the molecular docking method was examined by using the docking procedure of the innate ligand to the prepared target enzyme. Furthermore, the optimized rutin is docking to the prepared target enzyme using Molecular Operating Environment (MOE, 2016) version 10 program^35^. The default program settings were employed during the performance of docking process. The lowest-free binding energy docked poses for the examined ligand in the catalytic site of tyrosinase were used for further analysis. The structures of the examined inhibitors were presented (Fig. 1).

### Statistical analysis

The assays were performed in three independent experiments and the data are represented as mean value ± standard deviation. The statistical analyses were conducted with one-way ANOVA. The concentration of the examined sample which reduced the initial concentration by 50% (IC_50_ value) was determined using a linear regression of the developed data.

## Results and discussion

### Tyrosinase inhibition

**Figure 2:**
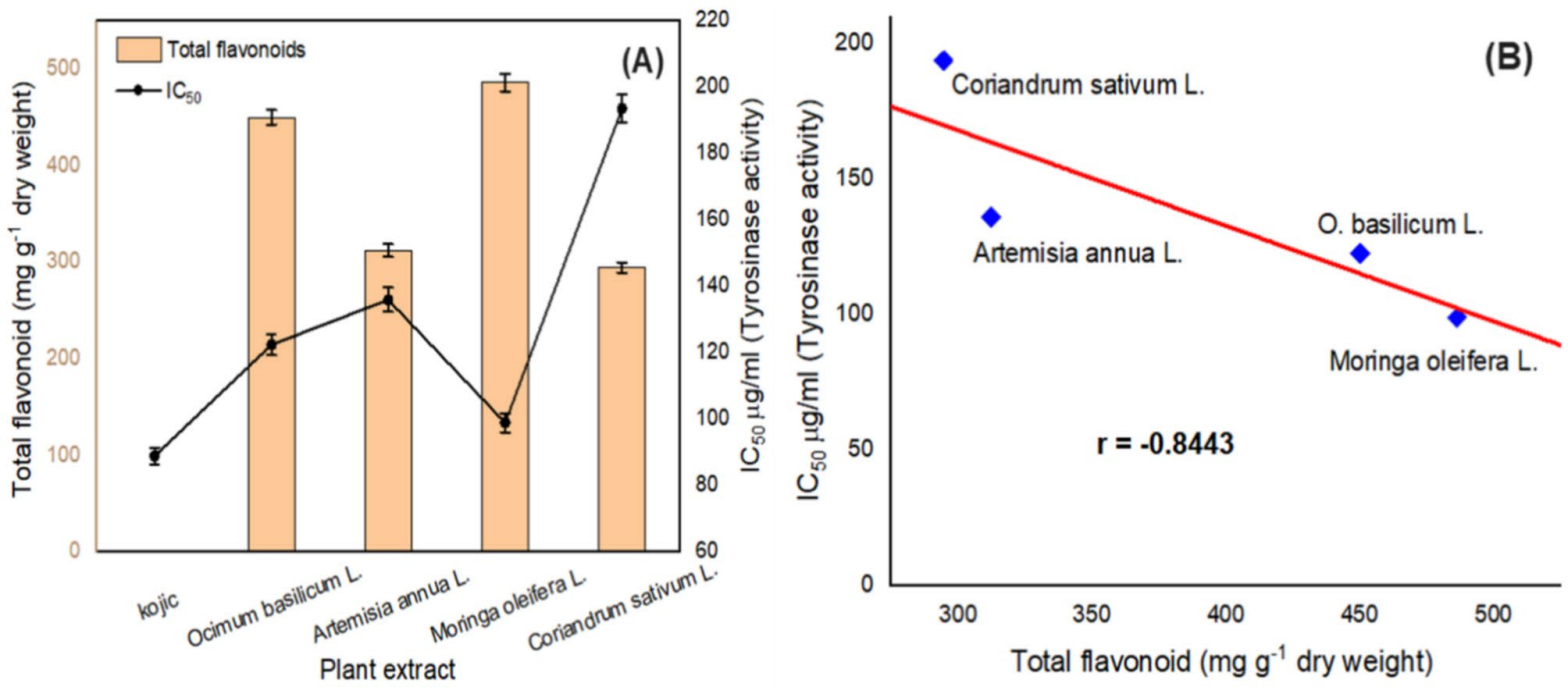
Tyrosinase inhibitory activity using the extract of different plants when _L_-DOPA used as substrate. (**A**) Representation of the total flavonoids in the extract of various plants and the matching IC_50_ of the inhibition for tyrosinase-diphenolase activity with respect to the concentration of inhibitor. (**B**) Representation of the correlation among the total flavonoids in the extract of the four tested plants and the activity of tyrosinase-diphenolase inhibition.

The tyrosinase inhibition using the extract of different plants and kojic acid as positive control has been examined with a fixed _L_-DOPA concentration and varying the inhibitor concentration. Linear regression has been developed by plotting tyrosinase activity inhibition versus the initial concentration of inhibitor^3,4^. A strong inhibition of tyrosinase-diphenolase activity (TYR-Di) has been determined by using all examined plants extracts. All examined extracts displayed a concentration-dependent inhibition toward the activity of TYR-Di. The developed data were expressed as the concentration of inhibitor which can inhibit 50% of tyrosinase activity (IC_50_) as illustrated in (Fig. 2A). The order of IC_50_ values for TRY-Di inhibition using *Moringa oleifera* L., *Ocimum basilicum* L., *Artemisia annua* L., and *Coriandrum sativum* L. were respectively as follow: 98.93, 122.49, 136.01, and 193.67 µg/ml, however, the IC_50_ values for TRY-Di inhibition using kojic acid was 88.92 µg/ml. The extract of *Moringa oleifera* is a promising potent inhibitor for the inhibition of TYR-Di when compared with the other plant extracts, based on the values of IC_50_. This may be ascribed to the high contents of flavonoids and phenolics constituents. Among the investigated plants, *Coriandrum sativum* L. displayed the lowest flavonoids contents, however, the *Moringa oleifera* L. showed the highest contents of flavonoid compounds.

A bivariate correlation (Pearson coefficient) has been employed for the determination of the relation among the total flavonoids in the plant extract and IC_50_ of TYR-Di inhibition (Fig. 2B). In this study, the correlation coefficient (r) was found to be – 0.8443 among the two variables which designates a good negative correlation. Hence, the TYR-Di inhibition may be due to the flavonoids in the plant extract^24,43^. Previous researchers have been described that the extract of different plants had a potent inhibitory effect on the tyrosinase activity^2,43–45^. A remarkable inhibitory effect of the *M oleifera* L. leaves extract on the tyrosinase activity have been reported to be due to a high content of flavonoid compounds^24,28^. Derivatives of kaempferol, luteolin, and quercetin reported as major flavonoid compounds which are predominant in the plant extract and exhibited a significant inhibition on the catalytic reaction of tyrosinase^5,28^.

### Type of tyrosinase inhibition

**Figure 3:**
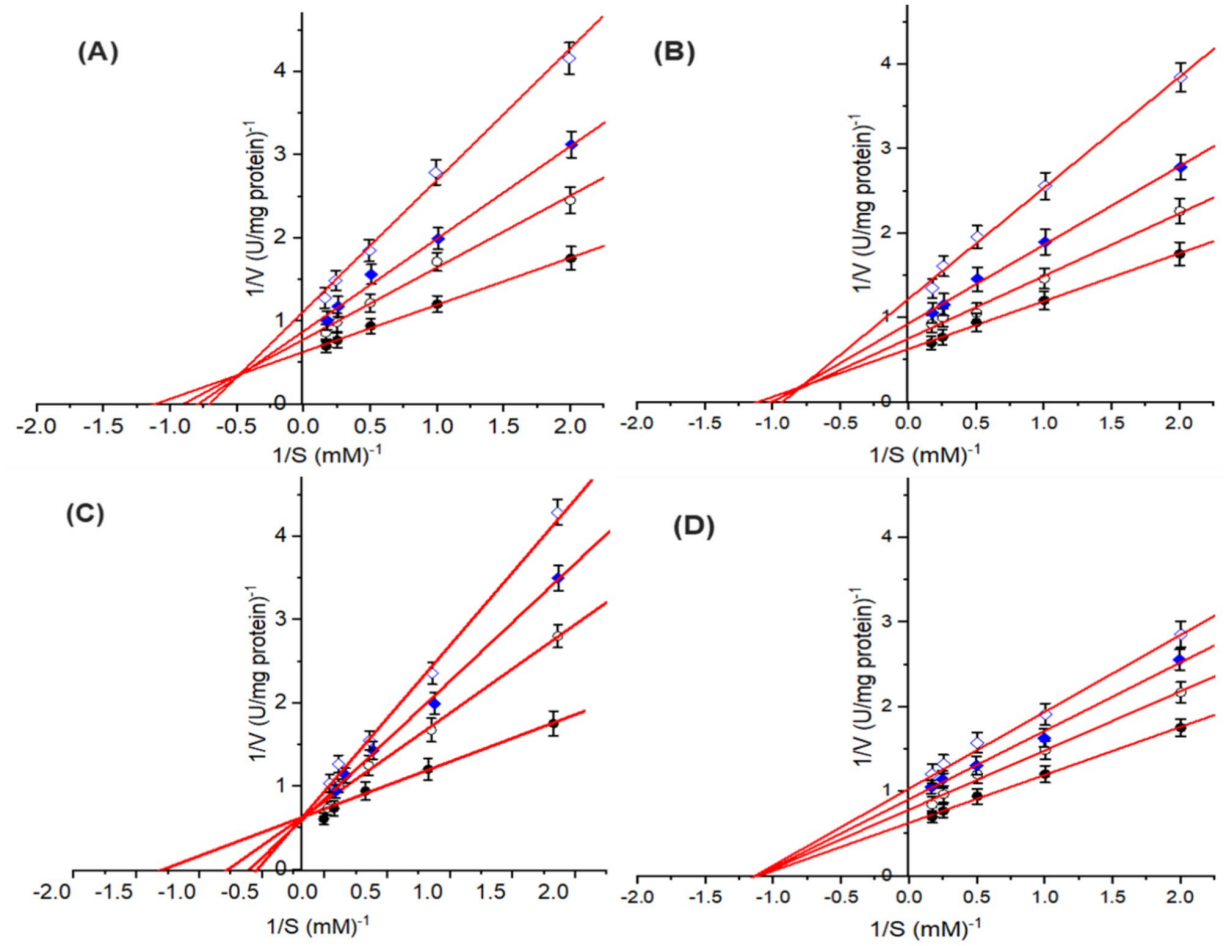
Tyrosinase inhibitory activity using the extract of *Ocimum basilicum* L. (**A**) and *Artemisia annua* L (**B**), *Moringa oleifera* L. leaves (**C**) and *Coriandrum sativum* L. (**D**) when l-DOPA used as substrate. Representation of the Lineweaver–Burk plots for tyrosinase-diphenolase activity inhibition in the absence of inhibitor () and in the presence of different concentration of inhibitor, namely 50 µg/ml (), 100 µg/ml (), and 150 µg/ml ().

To determine the inhibition type for *A. bisporus* TYR-Di, the potent extract of the examined plant parts was selected after performing interpolations based on the low IC_50_ value. The values of V_max_ and K_m_ were calculated from the Lineweaver–Burk plot using _L_-DOPA as substrate. The results revealed that the enzyme catalytic activity followed Michaelis–Menten kinetics. The V_max_ was 1.58 U/mg protein and K_m_ of 0.89 mM for the diphenolase activity of TYR using the dataset obtained in the absence of an inhibitor. At various inhibitor concentrations, the kinetics and type of TYR-Di inhibition were clearly varied according to the type and concentration of examined plant. The developed Lineweaver–Burk plots of the TYR-Di activity with exposure to various inhibitor doses showed that the inhibition was mixed type (i.e. in which the enhancement of inhibitor concentration triggered increase in the value of K_m_ and decreases in the V_max_ value) in the presence of the extract of *Ocimum basilicum* L., and *Artemisia annua* L. (Fig. 3A,B). A non-competitive type of inhibition on TYR-Di (i.e. in which the supplement of extra inhibitor triggered reduction in the value of V_max_ in the presence of the extract of *Coriandrum sativum* L. (Fig. 3D). However, the extract of *Moringa oleifera* L. leaves displayed competitive inhibition (i.e. in which the supplement of extra inhibitor triggered increment in the value of K_m_ and kept the value of V_max_ unaltered) on the TYR-Di activity. The values of V_max_ were distinctly reduced by 19.3, 30.2, and 40.7% in the presence of 50, 100, and 150 µg/ml of *Moringa oleifera* L. leaves extract with respect to control without inhibitor represent 100%, respectively. While the K_m_ values were sharply reduced in the range from 29.5 to 54.4% in the presence of inhibitor dose of 50–150 µg/ml, compared to the preparations without inhibitor (Fig. 3C). Similar results have been mentioned by^2,44–46^.

**Figure 4:**
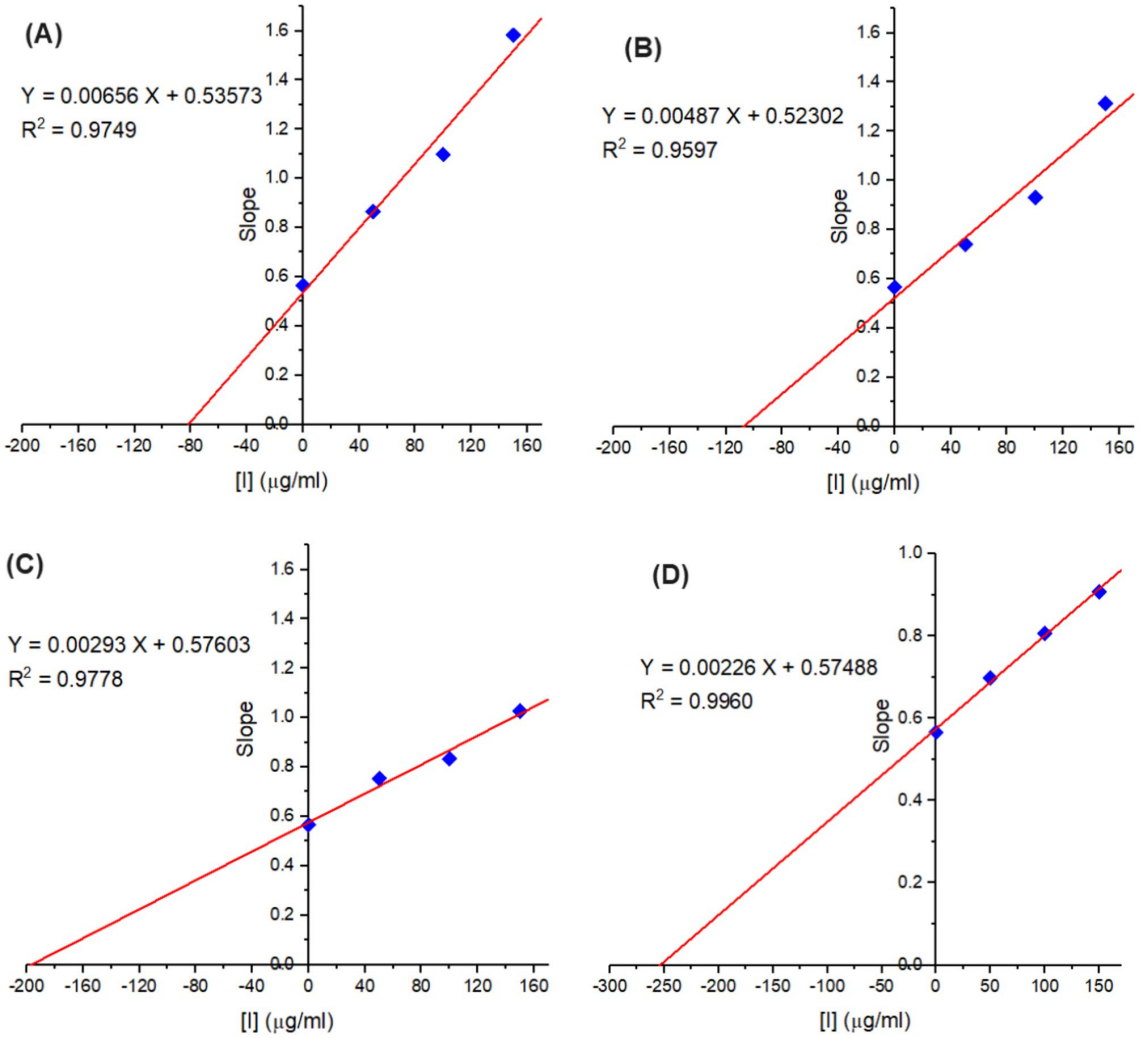
Plot of the slope from the Lineweaver–Burk plots versus different inhibitor concentrations which used to detect inhibition constants (K_i_) of tyrosinase by the extract of *Ocimum basilicum* L. (**A**), *Artemisia annua* L. (**B**), *Moringa oleifera* L. (**C**) and *Coriandrum sativum* L. (**D**). A linear regression fit was employed to determine the linear equation and regression coefficient.

**Figure 5:**
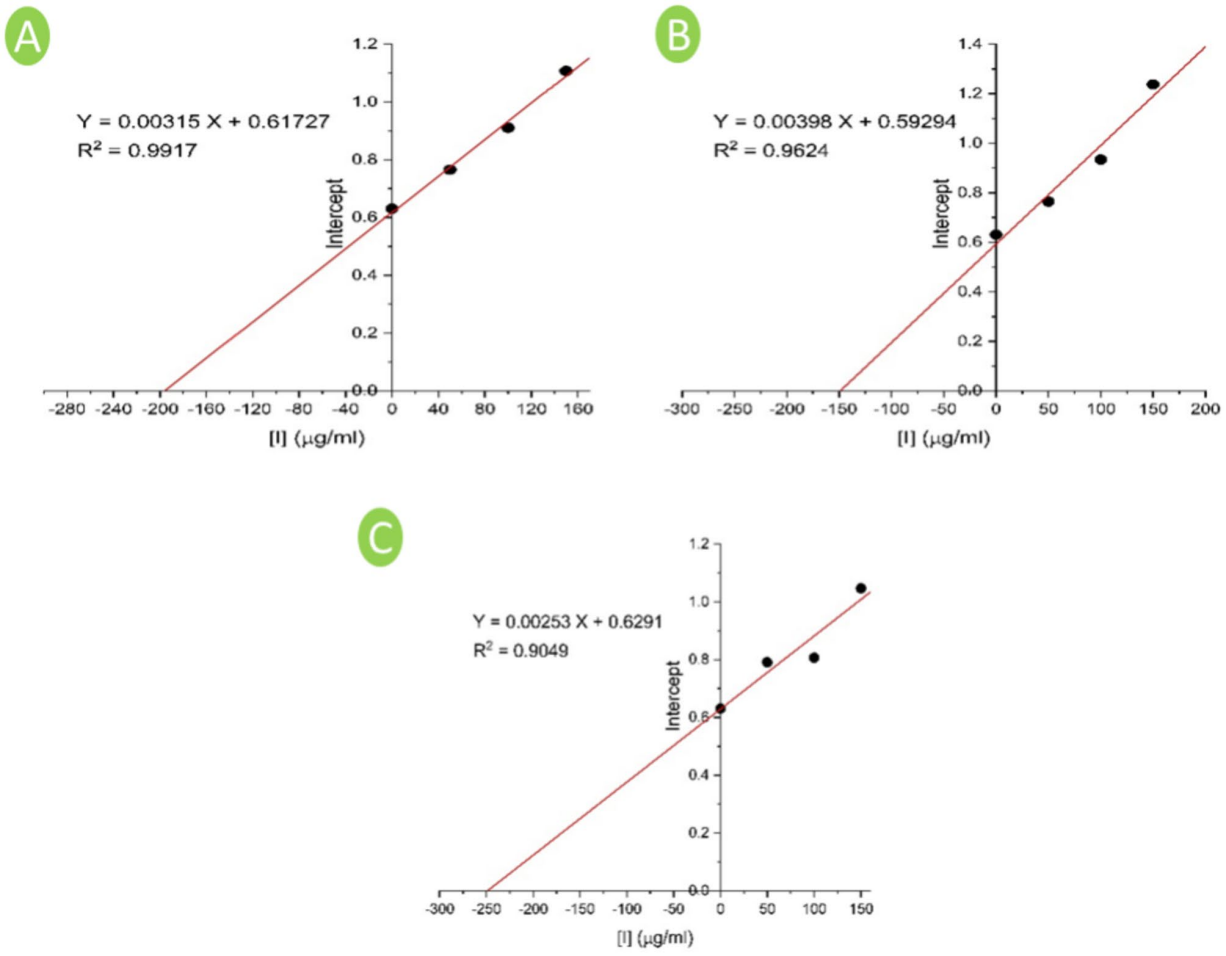
Plot of the intercept from the Lineweaver–Burk plots versus different inhibitor concentrations which used to detect inhibition constants (K_ii_) of tyrosinase by the extract of *Ocimum basilicum* L. (**A**), *Artemisia annua* L. (**B**), and *Coriandrum sativum* L. (**C**). A linear regression fit was employed to determine the linear equation and regression coefficient.

The mechanism of inhibition of the selected plant extract for the diphenolase activity of tyrosinase was further examined via the determination of the inhibition constants^43^. The (${\text{K}}_{\text{i}}^{\text{app}}$) which is the equilibrium constant of the inhibitor binding to the free tyrosinase and the (${\text{K}}_{\text{ii}}^{\text{app}}$) which is the constant for the binding of inhibitor for the enzyme–substrate complex were determined according to^28^. From the obtained results of the double reciprocal plots of Lineweaver–Burk, a secondary plot of the apparent slopes (K_m_/V_max_) and *y*-intercepts (1/V_max_) were performed against various inhibitor concentrations. The V_max_ values were clearly reduced, and K_m_ values were increased during the rise in the concentrations of the extract of *Ocimum basilicum* L. and *Artemisia annua* L. The results also revealed that the tested inhibitors (plants extract) could combine with the free enzyme and with the enzyme–substrate complex. The inhibition equilibrium constants (${\text{K}}_{\text{i}}^{\text{app}}$) of *Ocimum basilicum* L. and *Artemisia annua* L. on TYR-Di activity using _L_-DOPA substrate were respectively found to be 75, and 110 µg/ml (Fig. 4A,B). While the (${\text{K}}_{\text{ii}}^{\text{app}}$) of *Ocimum basilicum* L. and *Artemisia annua* L. on TYR-Di activity were 200, and 155 µg/ml when using _L_-DOPA substrate, respectively (Fig. 5A,B). In the previous circumstances, the values of ${\text{K}}_{\text{i}}^{\text{app}}$ were significantly lower than the values of ${\text{K}}_{\text{ii}}^{\text{app}}$, representing that the inhibitor displays as competitive-uncompetitive mixed-I type inhibitor^28,43,47^. This behavior indicates that the competitive influence was greater than the uncompetitive one, hence the free TYR inhibited more than the TYR-Di complex.

The inhibitory kinetics of the extract of *M. oleifera* L. leaves displayed a rise in the values of K_m_ and retained V_max_ unaltered indicating a competitive inhibition of the extract of *Moringa oleifera* L. leaves on the activity of TYR-Di using _L_-DOPA substrate (Fig. 4C). This inhibition type suggests that the extract can inhibit only the free enzyme. Hence, the value recorded for the ${\text{K}}_{\text{i}}^{\text{app}}$ constant (198 µg/ml) as well as the competitive type of inhibition proposed that the *Moringa oleifera* L. leaves extract contains a strong potent inhibitor on the TYR-Di activity.

The extract of *Coriandrum sativum* L. showed a sharp reduction in the V_max_ value, while the value of K_m_ was unchanged, indicating a noncompetitive inhibition of the TYR-Di activity. Additionally, the ${\text{K}}_{\text{i}}^{\text{app}}$ value was the same as the value of ${\text{K}}_{\text{ii}}^{\text{app}}$. (Figs. 4D, 5C). Therefore, the inhibitor can bind to both the free enzyme and the enzyme–substrate complex. From the previous results, it is significant that the extract of *M oleifera* L. leaves with a competitive inhibition type which is rarely observed amongst different enzyme inhibitors and has a potent specific inhibitor in the extract toward the TYR-Di, is a more promising TYR-Di inhibitor than other examined ones. The influence of the presence or absence of non-ionic surfactant on the TYR-Di inhibition by the extract of *M. oleifera* L. leaves was evaluated. The results revealed the inhibition percentage is not significantly reduced by using various concentrations of Triton X-100. Hence, the used surfactant cannot unmask the hidden TYR catalytic site and subsequently affirmed the absence of Pan-Assay Interfering Substances (PAINS) using the tested extract. These findings are well agreed with those obtained by^24^.

### Assessment of antioxidant activity and flavonoid isolation from *M. oleifera* L. leaves extract

**Figure 6:**
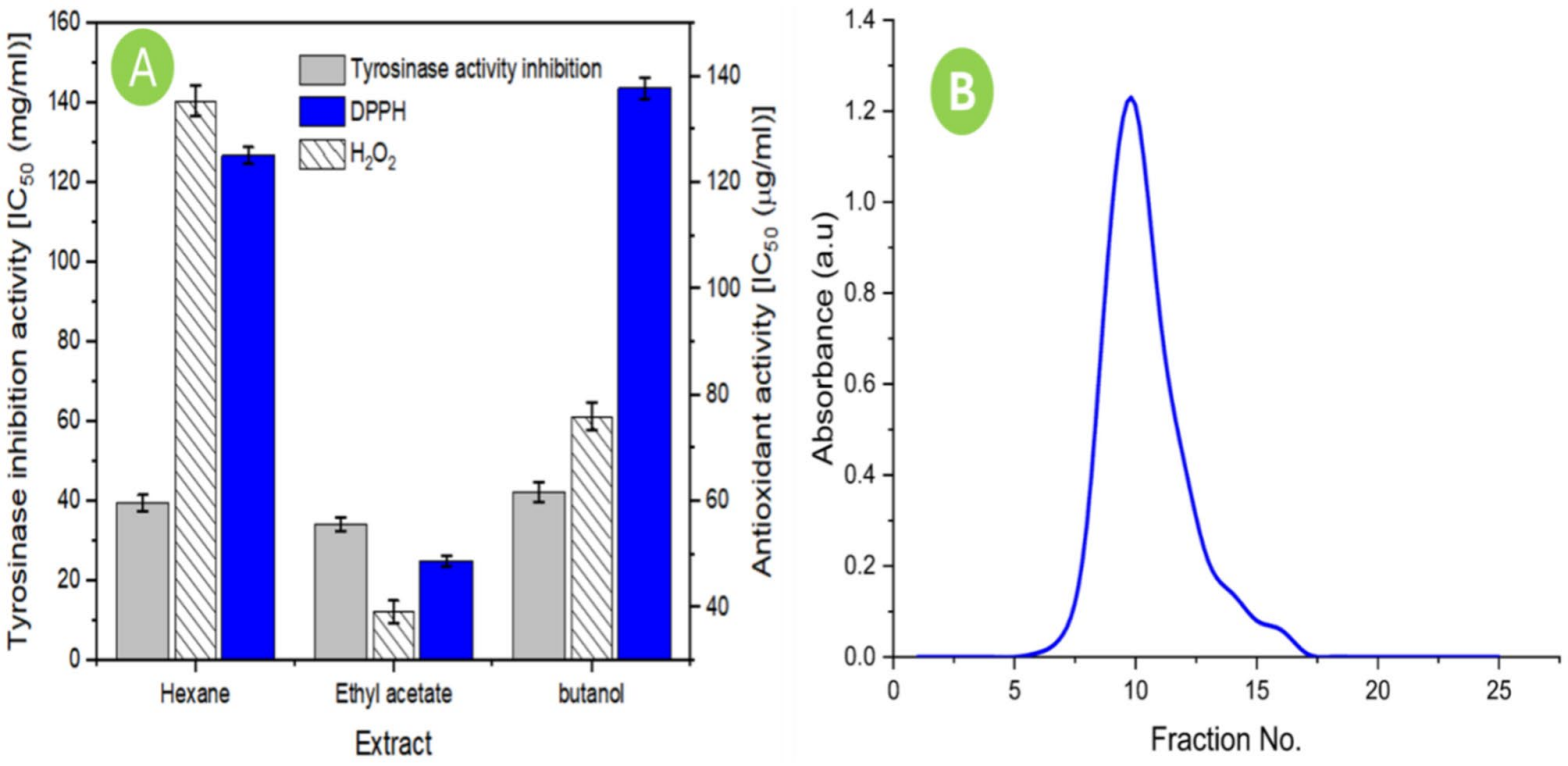
(**A**) The tyrosinase inhibitory activity represented in IC_50_ (µg/ml) and the antioxidant activity displayed in IC_50_ (µg/ml) of *Moringa oleifera* L. fractions. The IC_50_ designates the concentration of fraction which inhibits 50% (**B**) The absorbance spectrum at 510 nm of flavonoids fractions derived from leaves of *Moringa oleifera* L. for ethylacetate fraction.

The flavonoid constituents of *Moringa oleifera* L. leaf powder were fractionated using n-hexane, ethyl acetate, and n-butanol. The fraction of ethyl acetate for *M. oleifera* L. leaf powder displayed not only the maximum antioxidant activity for hydrogen peroxide and DPPH radicals, but also the lowest value for IC_50_ (34.3 µg/ml) of tyrosinase inhibition as shown in (Fig. 6A). Hence, the obtained fraction of ethyl acetate was employed for the flavonoid’s purification. The highest enzyme inhibition and antioxidant potentiality by using the ethyl acetate fraction of *M. oleifera* L. have been reported^24^, comparing to the other fractions. The promising antityrosinase and antioxidant activities of the ethyl acetate fraction of *M. oleifera* L. leaves may be attributed to its flavonoids constituents^46,48,49^. The antioxidant activity of rutin is linked to the presence of various chemical features including hydroxyl-, and oxo-functional groups, unsaturation, and catechol group that donate electron radical species^34^. After concentration in rotary evaporator, the developed flavonoids fractions exhibited a single peak at 510 nm (Fig. 6B), the fractions were then gathered in a tube and concentrated using rotary evaporator.

### Identification of the extracted flavonoids from *Moringa* oleifera leaves

**Figure 7:**
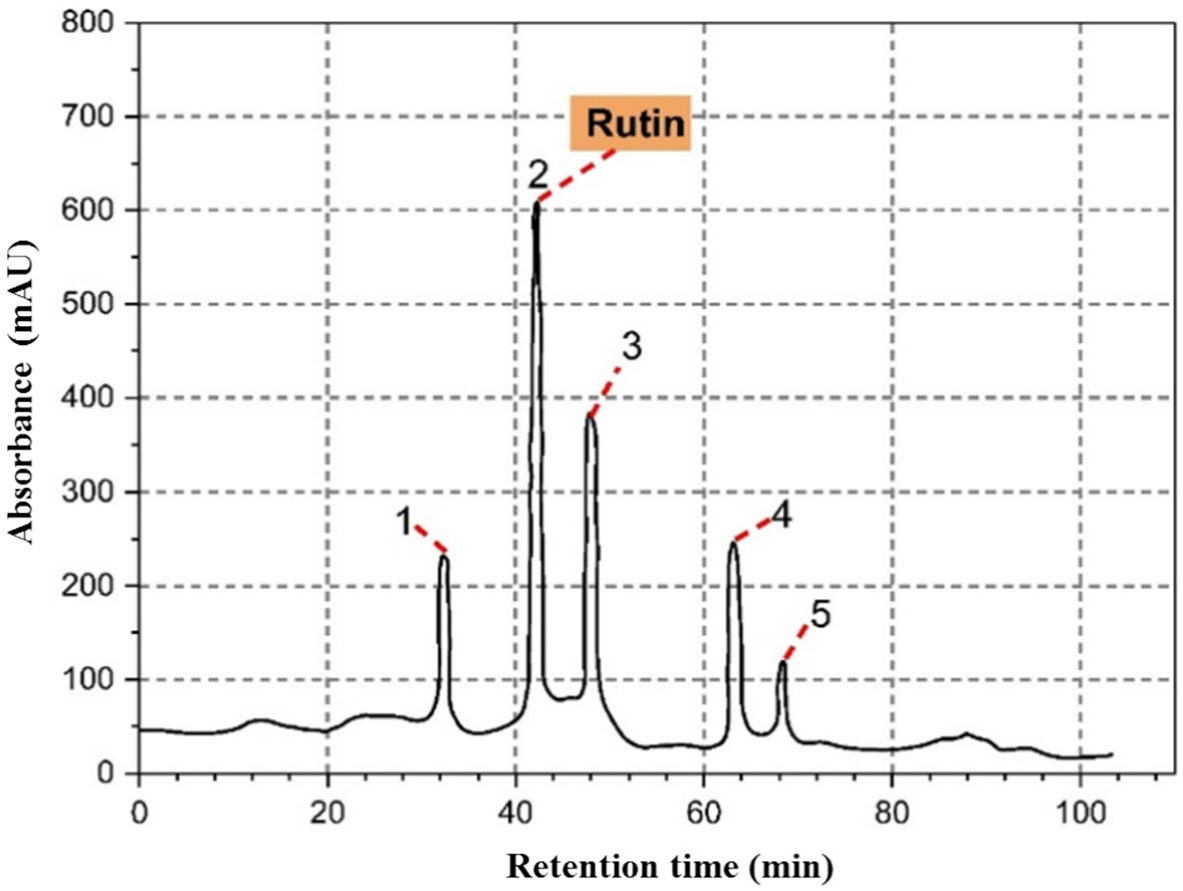
Representation of the HPLC chromatogram peaks of the crude *Moringa oleifera* L. leaves methanolic extract. The HPLC analysis was performed by injecting 20 µl of crude methanolic extract of leaves (10 mg/ml). The identity of the developed compounds was identified according to their retention time. The peaks # 1, 2, 3, 4 and 5 refers to the Gallic acid, Rutin, Kaempferol, Myricetin and Apigenin, respectively.

The HPLC chromatogram of the purified flavonoids in the extract of *Moringa oleifera* L. leaf powder is illustrated in Fig. 7 and Table S1. This chromatogram revealed the presence of multiple peaks, which were detected at various retention times. Five peaks were noticed with a 33.11, 41.86, 46.10, 64.11, and 68.51 min retention time. The chemical identity of the purified flavonoids was determined based on the comparison with the available authenticate sample. From the HPLC chromatogram, three major chemical components were determined namely, rutin, kaempferol, and myricetin at retention time 41.86, 46.10, and 64.11 min, respectively. The putative area for rutin, kaempferol, and myricetin was respectively found to be 38.56, 22.97, and 19.01%. There are additional peaks of anonymous compounds observed in the chromatogram. Hence, the results signify that rutin is the putative predominant flavonoid compound in the *M. oleifera* L. leaves extract with a primary antityrosinase activity which is highly comparable to a commercial inhibitor (kojic acid) for melanin biosynthesis. The HPLC-based chromatogram is clearly similar to that obtained from the examined samples of *M. oleifera* L. leaf extract, where rutin is uniquely major component in the investigated extract^31^; however, the glucosinolates, quercetin-3-*O*-glucoside, kaempferol, quercetin-3-acetyl-glucoside and other phenolic constituents are identified by HPLC analysis as the major component in *M. oleifera* L. leaves^24,50^. The quantity of extractible rutin from *M. oleifera* L. leaves is obviously enhanced by successive extraction using methanol^31^. On contrary, flavonoids compounds and their glucosides (not rutin) are identified as the predominant constituents in the *M. oleifera* L. leaves extract as reported by^26,50^.

### Chemical identity of putative rutin from *Moringa* oleifera

**Figure 8:**
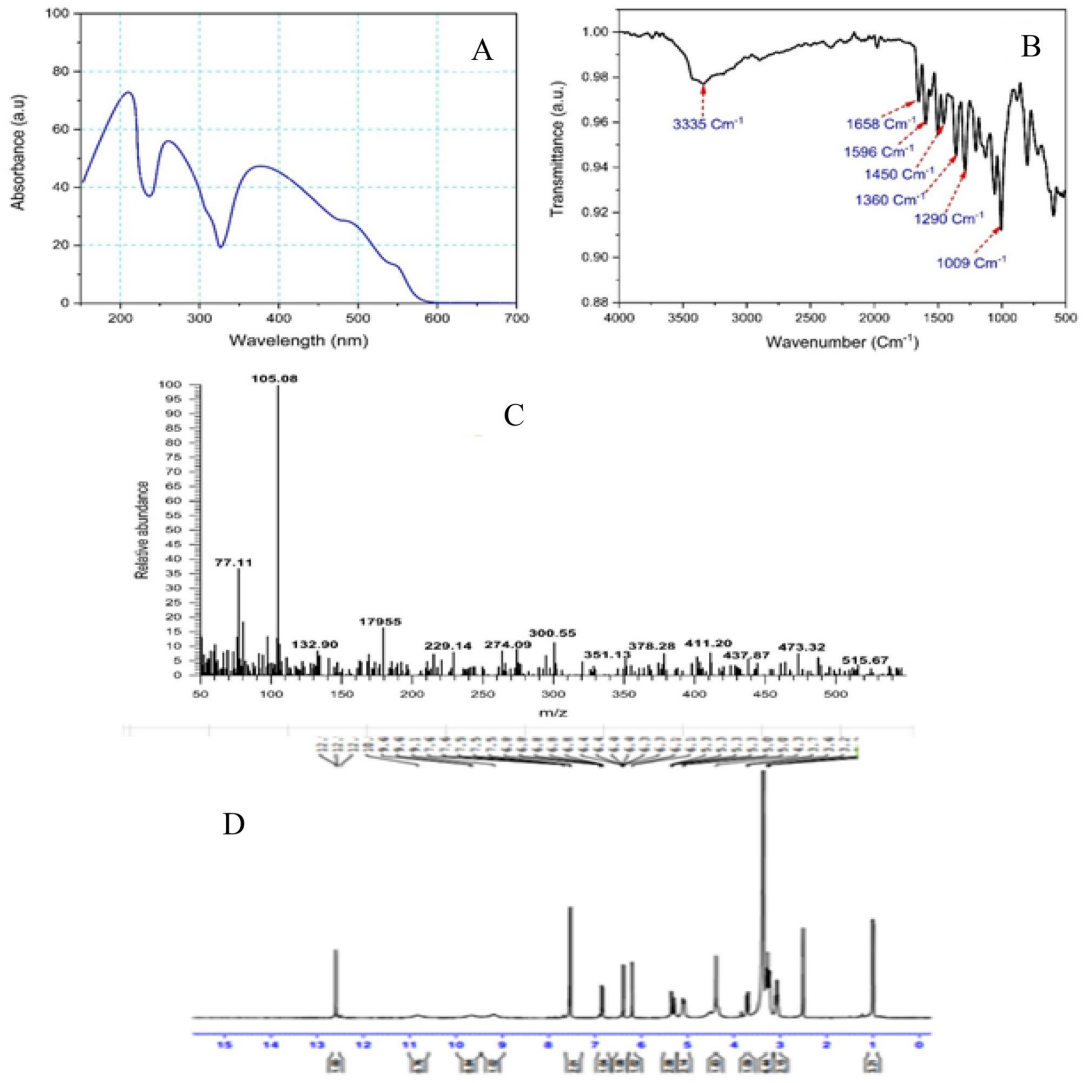
The **c**hemical analyses of the putative rutin from *Moringa oleifera.* UV-Spectra (**A**), FTIR (**B**) and LC–MS (**C**), and 1H-NMR (**D**), of the purified rutin from *Moringa oleifera*.

The ethyl acetate extract was subjected to column chromatography to isolate and purify the putative rutin. The flavonoid rutin is then chemically identified by UV–Vis, FTIR, mass spectrometry, and ^1^H NMR spectroscopic analyses. The putative rutin extracted from *M. oleifera* L. leaves displayed the λ_max_ wavelength at 215, 256, and 370 nm as revealed from the UV absorption spectrum (Fig. 8A) which are similar to the reference sample as reported by^51^. Hence, the developed absorbance peaks affirmed the structural identity of the putative sample as rutin.

The FTIR analysis was used to reveal the structure of rutin and is illustrated by Fig. 8B. From the FTIR spectrum, a broad absorption peak was observed at 3335 cm^−1^ which corresponds to the O–H overlapping stretching vibrations of the sugar and phenolic groups^29,52^. A peak with low intensity was determined at 1658 cm^−1^ was assigned for the C=O stretching vibration. The absorption peaks at 1596, 1450, and 1461 cm^−1^ related to the C=C stretching vibration of the aromatic ring^34^. The peaks appeared in the range of 1000–1360 cm^−1^ could be attributed to C–H, C–O–C, C–C, and C=O vibrations^53^.

The mass spectrum analysis enables the characterization of the unidentified bioactive compounds depends on the detection of the exact mass and the diagnostic parent and product fragment of the compound using MS/MS fragmentations^28,31,54^. Previous reports on the quantitative and qualitative identification of phenolic compounds in the crude extract of various plant extracts are performed using HPLC^28^, and GC–MS^54^, and liquid chromatography-mass spectrometry (LC–MS)^55^. The mass spectrum of the putative compound was tentatively characterized as rutin with a characteristic product fragment ion at 300.55 m/z and at 274 m/z (Fig. 8C). Similar results have been determined for the rutin from leaves extract of *Moringa stenopetala*^31^.

The ^1^H NMR spectrum of the purified rutin (Fig. 8D), was compared with the results from the literature. From the ^1^H NMR data, signals at δ 4–5.5 ppm of aliphatic -OH groups of rutinose were detected. Signals at δ 5.07–4.38 ppm were individually observed, hinting the presence of aliphatic CH of the rutinose units. Additionally, the peaks at δ 3–4 ppm were recorded as the residual signals of C–H of rutinose. The chemical peaks of aromatic OH groups were reported at δ 9–13 ppm; however, the aromatic CH resonances were noticed at δ 6–8 ppm. The ^1^H NMR spectrum affirmed the presence of resonances for rutinose moiety, aromatic and aliphatic CH and OH groups of sugar, and hence the substance is unambiguously identified as rutin when compared with typical in the literature data^34,53,56^.

### Molecular docking of tyrosinase inhibited by Rutin

The structure of tyrosinase is characterized by the H_2_L_2_ tetramer structure. The 2H subunits comprise 392 amino acid residues and have 2 Cu site, however, the 2 L subunits contains 150 amino acid subunits. In the 2H subunits, the first Cu ion showed interaction with the histidine residues (HIS^85^, HIS^61^, and HIS^94^). While the second Cu ions demonstrate a coordination with HIS^263^, HIS^259^, and HIS^296^. Previous studies confirmed that the inhibition of tyrosinase (monophenolase and diphenolase) activity depends on the capability of the tested compound to interact with the histidine residues in the catalytic sites^8,16^.

**Figure 9:**
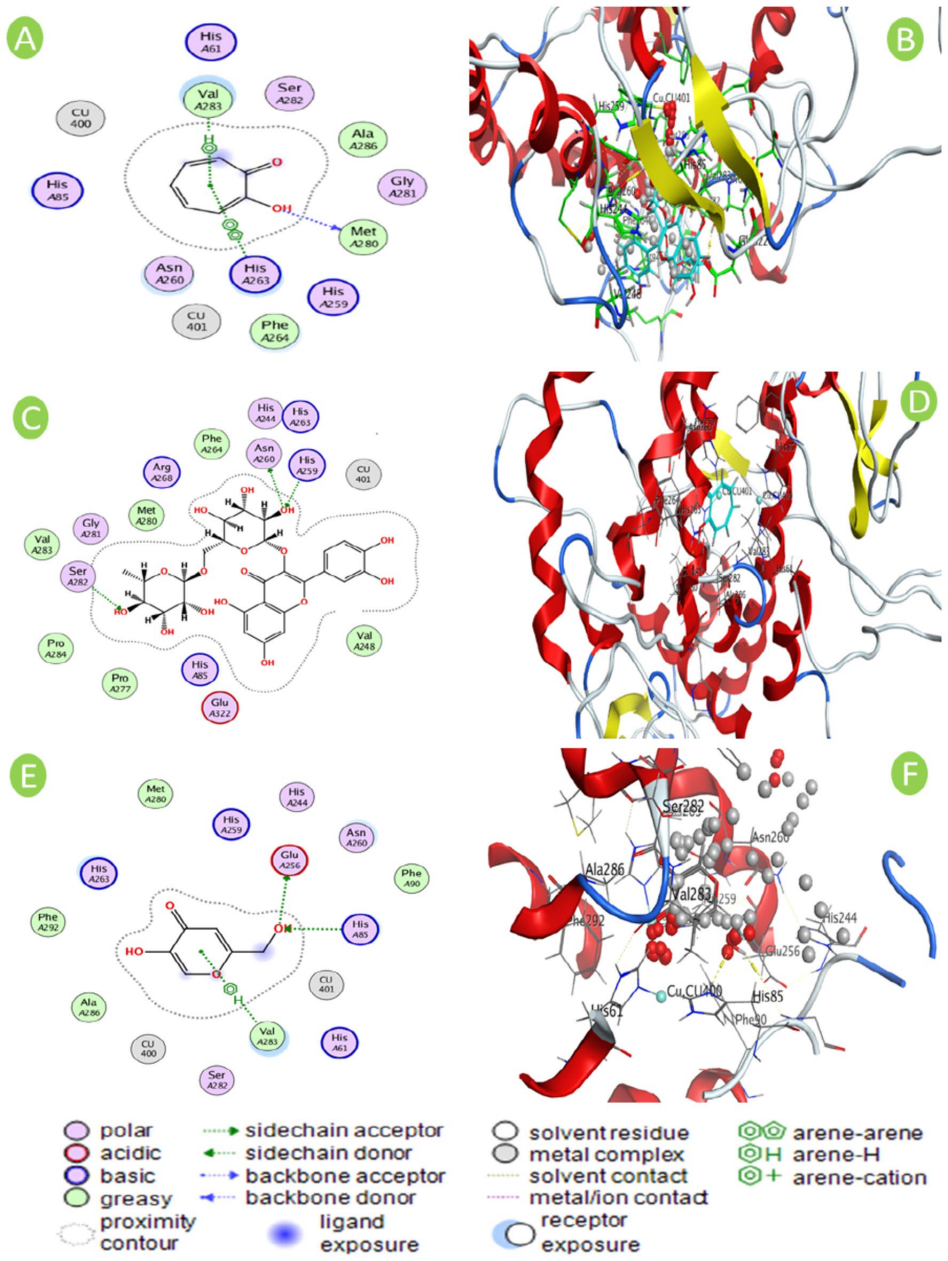
The conformation depiction of the interaction between different inhibitors and the binding pocket of tyrosinase from *Agaricus bisporus* (2X9Y) using Molecular operating environment (MOE) version 10 program software. The 3D visualization of interaction of amino acid residues in the active site of tyrosinase (2Y9X) with the co-crystallized tropolone (represented in cyan sticks) as positive inhibitor (**A**), rutin (represented in cyan sticks) inhibitor (**C**) kojic acid (**E**). The depiction of 2D interaction of amino acid residues in the active site of tyrosinase (2Y9X) with the co-crystallized tropolone as positive inhibitor (**B**), rutin inhibitor (**D**), and kojic acid (**F**). The structure of target protein was obtained from the last step of molecular docking. The computational simulation of rutin was performed in the active site, where located two copper ions (cyan spheres). The depiction of the molecular analysis was carried out using Molecular Operating Environment (MOE, 2015.10).

Bioactive compounds in the leaves extract of *M. oleifera* L., including the derivatives of kaempferol, rutin, luteolin, apigenin and quercetin have been depicted to inhibit the tyrosinase activity^24,28^. To evaluate the interactions and binding mode of the tested inhibitors with the amino acid residues in the tyrosinase catalytic site, the *in-silico* molecular docking analysis were performed using the crystallographic structure of the target protein for *A. bisporus* (PDB code: 2Y9X) with native inhibitor (tropolone). Validation of the molecular simulation was performed by re-docking the inherent ligand to tyrosinase (Fig. 9A,B) and then the developed rutin is docking to the target protein (Fig. 9C,D) using Molecular Operating Environment (MOE) version 10 program tools. The preparation of the target protein generates the active sites without inherent ligand, hence the pockets of enzyme active sites with its accessible amino acids residues becomes available for the docking simulation^5,8^. In the developed docked complexes, the bonding interaction and the binding energy values were used to predict the best docked position of the investigated compound against tyrosinase^5,8^. The validation of the simulation process was reached when the RMSD (root mean square deviation) value is lower than 3 Å. The re-docking of the co-crystallized native ligand with the 2Y9X tyrosinase displayed RMSD of 2.40 Å and RMSD of 2.29 Å for kojic acid, while the docking of rutin to 2Y9X tyrosinase was 1.38 Å (Table 1), indicating the validity of the molecular simulation process^5^.

**Table 1:** Binding energy, RMSD and hydrogen bond of various ligand against tyrosinase from *Agaricus bisporus.*

Protein	Ligand	BE (kcal/mol)	RMSD (Å)	Amino acid residue
Tyrosinase from *Agaricus bisporus* (2Y9X)	Rutin	− 7.75	1.38	HIS^259^, ASN^260^, SER^282^
	Tropolone	− 5.28	2.40	MET^280^, VAL^283^, HIS^263^
	Kojic acid	− 4.69	2.29	HIS^85^, GLU^256^, VAL^283^

* BE (Binding energy), RMSD (root mean square deviation).

The bond strength, stability, and affinity of the examined compound against the target protein depends on the value of binding energy^5,8,57,58^. The lower value of the binding energy, the higher bond strength, stability, and affinity of the examined compound against the target protein, were resolved^5,18,34,59^. The docking pose of rutin against 2Y9X-tyrosinase with the lowest energy was selected for further studies and it was found to occupy the same position of the inherent ligand. Hence, rutin is efficaciously binds to the active site of tyrosinase. The value of the binding energy for the interaction between the tropolone ligand and tyrosinase was − 5.28 kcal/mol, however the binding energy value of rutin and tyrosinase was − 7.75 kcal/mol (Table 1). Additionally, the energy of binding for kojic acid against tyrosinase protein was − 4.69. The docking score (S) showed that rutin compound is able to tightly bind to the tested enzyme (2Y9X) with efficaciously inhibition for the target protein tyrosinase.

The hydrogen bonds are visualized in the MOE program software. On contrary, the aromatic interactions, hydrophobic interactions, Van der Walls interaction, and ionic bonds cannot be observed in the output data of MOE program, however, they still affect the binding energy of the ligand in the active site of tyrosinase. Due to the presence of –H and –OH groups in rutin, hydrogen bonding is the most observed in the docking process. The possible docking interaction mode of rutin within the tyrosinase active site is illustrated in Fig. 9. The hydrogen bonds formed between the hydroxyl groups of rutin and the plausible residues in the core of active site for tyrosinase namely, ASN^260^, HIS^259^, and SER^282^. While, the π-π bond was observed among the HIS^263^ in the tyrosinase active site and the tropolone (inherent ligand) as positive inhibitor (Fig. 9B). The kojic acid inhibitor could bind with the amino acid residues in the tyrosinase active site namely, HIS^259^, ASN^260^, SER^282^ as shown in Fig. 9E,F. The strength of hydrogen bond by rutin was found to be 0.8–4 kcal/mol and reversible. The molecular simulation model clearly revealed that rutin obtained from the leaves extract of *Moringa oleifera* L. is able to strongly inhibit tyrosinase via binding to the amino acid residues in the catalytic site, and hence reduces the melanogenesis. The obtained simulation results harmonized well with^1,18,21^ who mentioned that rutin is successfully bound to only the amino acid residues in the enzymatic cavity; however, other docking search performed by^25,60^ detected the ability of rutin to coordinatively bind to Cu ion in the catalytic site.

## Conclusion

The hydro-alcoholic extract of *Moringa oleifera* L. leaves displayed the highest potent anti-tyrosinase activity in a dose-dependent manner using _L_-DOPA as substrate, compared to the those of other plants extract. The inhibition kinetics, types, and mechanism were clearly varied based on the type and concentration of the plant’s extracts. A mixed inhibition type was determined by the *Ocimum basilicum* L and *Artemisia annua* L extracts, while the *Coriandrum sativum* L extract displayed a non-competitive type of inhibition. The observed competitive inhibition and low ${\text{K}}_{\text{i}}^{\text{app}}$ value using the extract of *Moringa oleifera* L. leaves, implied that the extract can specifically inhibit the TYR-Di complex. Hence, the *M. oleifera* L. leaves extract contains strong potent inhibitors. The ethyl acetate extract of the *Moringa oleifera* L. leaves showed the highest anti-tyrosinase and antioxidant activities which was linked to its high flavonoid constituents as verified by HPLC chromatogram. The putative predominant rutin compound was isolated and purified using column chromatography, and then chemically identified by different spectroscopic analyses including UV spectrophotometer, FTIR, mass spectrum, and ^1^H NMR. The docking simulation results revealed the tight binding of rutin against the 2Y9X amino acid residues in the tyrosinase catalytic site and hence efficaciously inhibits tyrosinase with respect to its promising application in cosmetic industries. Further ongoing searches are required to evaluate the safety and efficiency of natural rutin in hyperpigmentation disorders via in vivo approach and to develop new agents for pigmentary disorders.

### Supplementary Information

Supplementary Table S1.

## Data Availability

All the data are provided within the manuscript.
