# Supplementary material for: Rutin of Moringa oleifera as a potential inhibitor to Agaricus bisporus tyrosinase as revealed from the molecular dynamics of inhibition
Source: Sci Rep. 2024 Aug 29;14:20131. doi: 10.1038/s41598-024-69451-y (PMC11362471; doi:10.1038/s41598-024-69451-y)
Supplement: Supplementary file 1 — Supplementary Table S1. [file 41598_2024_69451_MOESM1_ESM.docx]

Table S1: Compound identification of the methanolic extract of *M. oleifera* L using HPLC data based on their retention times.

| **Peak No.** | **Retention time (min)** | **[M-H]^-^ (m/z)** | **Expected compound identification** | **Reference** |
| --- | --- | --- | --- | --- |
| 1 | 33.11 | 88.3 | Gallic acid | [24, 28, 51] |
| 2 | 41.86 | 195.9 | Rutin |  |
| 3 | 46.10 | 123.1 | Kaempferol |  |
| 4 | 64.11 | 102.8 | Myricetin |  |
| 5 | 68.51 | 41.0 | Apigenin |  |
